# Insights into the morphology and molecular characterisation of glacial relict *Eurytemoralacustris* (Poppe, 1887) (Crustacea, Copepoda, Calanoida, Temoridae)

**DOI:** 10.3897/zookeys.864.34259

**Published:** 2019-07-15

**Authors:** Łukasz Sługocki, Anna Rymaszewska, Lucyna Kirczuk

**Affiliations:** 1 University of Szczecin, Faculty of Biology, Szczecin, Poland University of Szczecin Szczecin Poland; 2 University of Szczecin, Center of Molecular Biology and Biotechnology, Szczecin, Poland University of Szczecin Szczecin Poland

**Keywords:** Brackish water, crustaceans, genetics, lake, rare species, zooplankton

## Abstract

*Eurytemoralacustris* (Poppe, 1887) is a stenothermic glacial relict whose narrow environmental requirements make it an indicator species for good ecological conditions. The primary threats to this species are eutrophication and global warming. Many authors have described *E.lacustris* in taxonomic keys; however, its morphological description is unsatisfactory. Therefore, in this study, we aimed to review morphological characteristics of *E.lacustris* that were previously undescribed in the literature and to provide the molecular characteristics based on the two conservative mitochondrial genes: cytochrome *c* oxidase I (*COI*) and cytochrome *b* (*cytb*). The new record of *E.lacustris* indicates that it is a more widespread species than previously hypothesized. Width-to-length ratio of the last female endopod segment of legs indicates variation among the widely distributed species of the genus in Europe (i.e., *E.lacustris*, *E.velox* (Lilljeborg, 1853), and *E.affinis* (Poppe, 1880)). We also found variability of number of setae on the second segment of male endopod. Furthermore, our analysis confirms the occurrence of species in different than exclusively freshwater habitats.

## Introduction

The marine, estuarine, and freshwater genus *Eurytemora* is represented in Europe by eight species (22 worldwide) (Błędzki and Rybak 2016). Recently, Alekseev and Souissi (2011) described a new species, *Eurytemoracarolleeae*, from the North American waters, which is invasive in European waters. Depending on the salinity of the water, *Eurytemora* shows highly evolvable traits (Lee 1999; Souissi et al. 2016). Most studies on *Eurytemora* concerns estuarine clade *Eurytemoraaffinis* (Poppe, 1880), which presents adaptations to freshwater habitats. The large morphological plasticity of *Eurytemora* in relation to its habitat can lead to confusion regarding the identification of the species. In contrast to the widely distributed species such as *E.affinis*, in Europe, other species of the genus are also present that represent relicts of *Eurytemora* on a global scale.

*Eurytemoralacustris* (Poppe, 1887) is a glacial relict that evolved from a marine ancestor in the ancient Holocenic Ancylus Lake into an exclusively freshwater species (Ekman 1922; Segerstråle 1957). Distributions of crustacean glacial relicts are restricted to the North European and North American lakes in regions that were covered by water after the ice age. This stenothermic copepod is restricted to lakes that are deeper than 30 m and where oxygen concentration in the hypolimnion is higher than 1 mg L^–1^ (Weiler et al. 2003; Kasprzak et al. 2005). *E.lacustris* is a dioecious and perennial species, unable to produce resting eggs (Kiefer 1978). Therefore, dispersion of the species is restricted mainly to the connected lakes (Maier et al. 2011). Its narrow environmental requirements make it an indicator species for waters with low trophy and good ecological condition (Karpowicz and Kalinowska 2018).

During the 20^th^ century, environmental impact resulted in the decline of the population of *E.lacustris*, which made the species more difficult to obtain than before (Maier et al. 2011; Vezhnovets et al. 2012). *Eurytemoralacustris* was recorded from eight Norwegian lakes (Spikkeland et al. 2016), five Lithuanian lakes (Arbačiauskas and Kalytytė 2010), five Latvian lakes (Paidere et al. 2011), two Belarusian lakes (Vezhnovets et al. 2012), and Russian Ladoga Lake (Avinsky et al. 2006). Lakes of Sweden and Finland probably have many lakes in which *E.lacustris* occur; however, the latest reports on this topic was published at the last century (Silfverberg 1999; Northcote and Hammar 2006). Formerly, *E.lacustris* was recorded from several German lakes, which at present is absent from some of them (Maier et al. 2011). Defaye and Dussart (2002) also recorded *E.lacustris* from the Danube and Volga basins and from North America (North Western Territories, Massachusetts). The presence of *E.lacustris* in Polish lakes has not been reviewed, but its distribution could be similar to the known distribution of malacostracan glacial relicts, which is also becoming less frequent in Polish lakes (Żmudziński 1990). Reports on the occurrence of *E.lacustris* in Poland mainly refer to North Eastern and Northern Poland (Lityński 1925; Czeczuga 1960; Karpowicz and Górniak 2013; Karpowicz and Kalinowska 2018) and rarely refer to Western Poland (Sługocki and Czerniawski 2018). A recent report from the Great Masurian Lakes (North Eastern Poland) showed high abundance of *E.lacustris* in three lakes, presumably as result of improvement of their trophic conditions (Karpowicz and Kalinowska 2018). The primary threats to this species are eutrophication and global warming (Weiler et al. 2003), and Belarusian, Lithuanian, (Vezhnovets et al. 2012), Estonian (Lilleleht 1998), and Norwegian (Kålås et al. 2010) red lists assessed *E.lacustris* as an endangered or vulnerable species.

*Eurytemoralacustris* was found beyond the reach of the glaciation ice sheet, which suggests that this species could not be considered as a typical postglacial relict (Spikkeland et al. 2016). Records of *E.lacustris* outside the glaciation area might have resulted from posterior colonization to new localities or due to lack of proper identification. The lack of resting eggs and its environmental requirements makes dispersion of this species very difficult. Therefore, records outside their natural habitat should be verified by morphological and molecular analyses.

Despite the fact that many authors have identified the species in taxonomic keys (Poppe 1887; Sars 1895; Dussart 1967; Einsle 1993; Błędzki and Rybak 2016), the morphological description of *E.lacustris* is unsatisfactory. Recent studies on the genus *Eurytemora* show that morphological techniques have great potential in copepod taxonomy (Lajus et al. 2015). In this article, we supplement the knowledge about this species with incompletely known characters, including legs morphology and the characteristics of the feeding appendages. The connection of morphological and molecular analysis allows to determine the proper taxonomic status of the specimens under study. The DNA barcoding methods, using amplification of the selected genes of the mtDNA, are commonly used to identify species of copepods. These gene sequences are used to distinguish between sibling species, identify species based on the remains of organisms, but they are also successfully used to describe unknown species. In molecular analysis, gene coding cytochrome *c* oxidase I (*COI*) and gene coding protein or complex III in oxidative phosphorylation (*cytb*) are used for the identification of species (Merritt et al. 1998; Costa et al. 2007; Thum and Derry 2008; Thum and Harrison 2008; Sukhikh et al. 2013; Blanco-Bercial et al. 2014; Gasmi et al. 2014). Such integrative studies using *COI* sequences and morphology of species were applied during the study of *E.carolleeae* (Vasquez et al. 2016) and *E.affinis* (Lee and Frost 2002). The use of both molecular and morphological analyses for the population of *E.lacustris* will lead to a better understanding of the population of *E.lacustris* and its natural history. So far, the molecular analysis of *E.lacustris* refers to several individuals from the Baltic Sea (GenBank; Alekseev and Sukhikh, unpublished), which is not a typical habitat for *E.lacustris*. Our study of the relict population of *E.lacustris* at Lake Cieszęcino in Poland is the first report from the lake and the first from Poland based on both morphology and molecular analysis of *COI* and *cytb* genes, which are commonly used for barcoding. These studies provide basis for further analysis of intraspecific diversity of the populations of *E.lacustris*. Therefore, in this study, we aimed to determine the morphological and molecular characteristics of *E.lacustris*.

## Materials and methods

**Figure 1.**
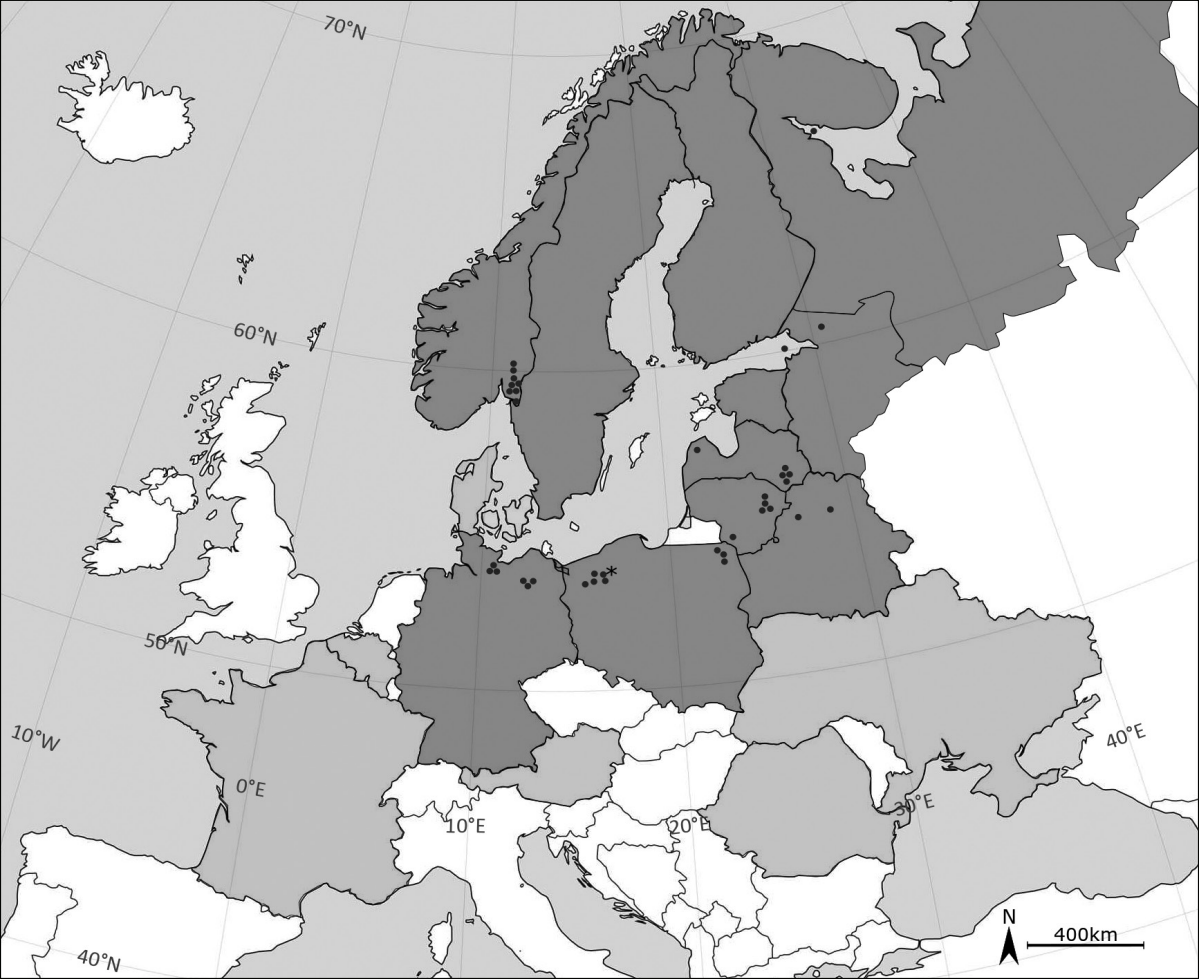
Distribution map of *Eurytemoralacustris*. Dark grey colour indicates present distribution of *E.lacustris*. Light grey colour demonstrates countries in which occurrence of this species is uncertain. Black dots indicate records in 21^st^ century, asterisk indicates a new record of *E.lacustris* in Lake Cieszęcino (Poland). The map does not include recent records in Volga basins (Popov 2011) and North America (Dussart and Defaye 2002).

Specimens of *E.lacustris* were obtained in September 2017 from Lake Cieszęcino (53°55'41.7"N, 16°49'29.6"E) (Fig. 1), north western Poland at an altitude of 154 m a.s.l. The lake has a surface of 102 ha; its deepest point reaches 39 m. The lake’s catchment area is 18.2 km^2^, of which 80% are forests and semi-natural areas (Corine Land Cover 2012). Anthropogenic and agricultural areas cover less than 1% of the catchment area, and the rest are wetlands.

Sampling station was set up at the deepest point, based on bathymetric maps (Jańczak 1996). In order to collect the specimens, a zooplankton net (mesh size 100 µm, d = 20 cm) was towed vertically from the bottom to the surface. Concentrated samples were poured into a 110-mL tube and fixed in a buffered 4% formalin solution (for morphological purposes), and a second sample was fixed in 70% alcohol (for genetic purpose). Zooplankton was analysed in plankton chambers using a Nikon Eclipse 50i microscope (Japan) and a Zeiss Primo Vert reverse microscope (Germany). The species was identified using taxonomic keys (Sars 1895; Einsle 1993; Błędzki and Rybak 2016).

Body size of the specimens was measured without caudal setae. Appendages were dissected using glycerine as the dissecting fluid, and measured under a microscope at 400 × magnification. Morphological description is based on adult female specimens. In two cases, the morphology of males is also mentioned (if so, it is emphasized). Specimens are stored at the collections of the University of Szczecin, Poland. Each variable was measured from digital photographs, using the software ToupView (ToupTek Photonics, China). The significance of differences in size between sexes was calculated using nonparametric Mann-Whitney *U* test (Statistica 12, StatSoft). For molecular analysis, individuals were transferred to PBS tubes (phosphate buffered saline) (n=80), followed by DNA isolation using the ready-made Tissue Genomic Extraction Mini Kit (with Proteinase K, Genoplast). Until the analyses were performed, the DNA was stored in a freezer (−70 °C).

Polymerase chain reaction (PCR) was performed twice for each specimen: amplification of the cytochrome *c* oxidase subunit I gene (*COI*, *cox1*) and *cytb*. Both genes are located in mtDNA and belong to the group of conservative genes that allow species identification. PCR amplification was conducted using the following primers: COIF-PR115 and COIR-PR114 for *COI* (Folmer et al. 1994) and UCYTB151F and UCYTB272R for *cytb* (Merritt et al. 1998). The results of PCR amplification were visualized by performing electrophoresis with 5 μL sample each in 1.5% agarose gels with GPB Gold View Nucleic Acid Stain (GenoPlast, Biochemicals, Poland).

Sequential analysis was performed for all samples. Sequencing was performed in Macrogen Europe (the Netherlands) with the same sets of primers that were used to obtain amplicons. The results were analysed using the Finch TV, BLAST, and Mega 7 software. Phylogenies were constructed using the Minimum Evolution method algorithm with Tamura–3-parameter model. A 1000-replicate bootstrapping was performed to obtain a measure of robustness of tree topology. *COI* and *cytb* sequences of *E.lacustris* have been reported to the GenBank (*cox1*: MH316160, MH316161; *cytb*: MH316162–MH316164). Due to the lack of data concerning deposited type specimens (Poppe 1887) genetic and morphological analyses were difficult to perform. Therefore, morphological traits that are described in this article based on specimens from population (Lake Cieszęcino) for which molecular characterization was performed.

## Results

### Morphology

**Figure 2.**
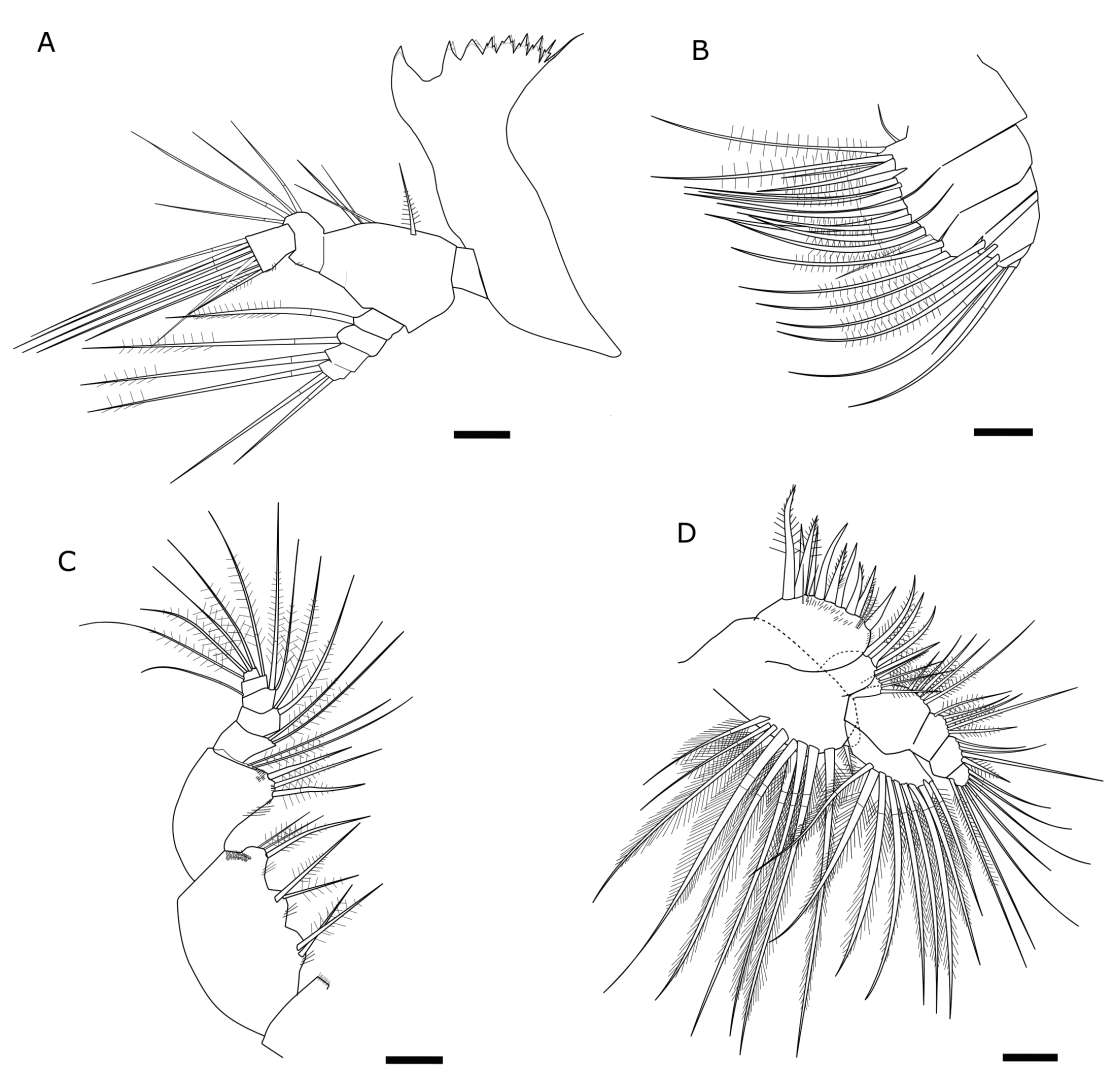
Feeding appendages of *Eurytemoralacustris***A** mandible **B** maxilla **C** maxilliped **D** maxillule. Scale bars: 20 µm.

Length of 40 adult specimens (20 females and 20 males) ranging from 1.274 to 1.483 mm. No significant differences in the body size between the two sexes (p > 0.05). Antennules of 24 segments with variable setation. Male right antennule with 21 segments. Most segments with two and more setae provided with one aesthetasc and distal segment with six setae and one aesthetasc. Antenna biramous, composed of two-segmented protopod, two-segmented endopod and seven-segmented exopod. First exopod segment with one seta, second segment with three setae, third to sixth segments with one seta each, seventh (distal) segment with four setae. First endopodal segment with two setae, second with nine setae laterally, and seven setae at distal end. Mandible (Fig. 2A) with sharp ventralmost tooth that corresponds to the largest tooth of the mandible. Size and shape of teeth (pars molaris) varied from a molar shape to acute teeth. Trident-shaped teeth from third to seventh. Distal tooth long and thin (spike-like), at least two times longer than seventh tooth. Coxa heavily sclerotized medially; basis with four setae. Exopod four-segmented with one seta on the first segment, second, and third segment and three setae on the fourth segment. Endopod two-segmented, with 4 and 11 setae. Maxilla (Fig. 2B) uniramous, precoxa with two endites, first endite with five setae, distal endite with three setae; coxa with two endites bearing three setae each; 5-segmented endopod including basal endite with one long, and four short segments bearing one or two long distal setae. Maxilliped (Fig. 2C) with syncoxa consisting of three lobes with respectively two, two, three setae; basis with five setae (two on a distal medial lobe). Endopod five-segmented with 2, 2, 2, 2, 4 setae. Maxillule (Fig. 2D) composed of precoxa with medial arthrite bearing nine strong spines; coxa with elongated endite bearing four setae and outer outgrowth with six strong sub-equal in length setae and three thin setae. Basis composed of basal endite, one-segmented exopod with nine long subequal setae and four-segmented endopod bearing 5–5–4–7 (distally) long setae.

**Figure 3.**
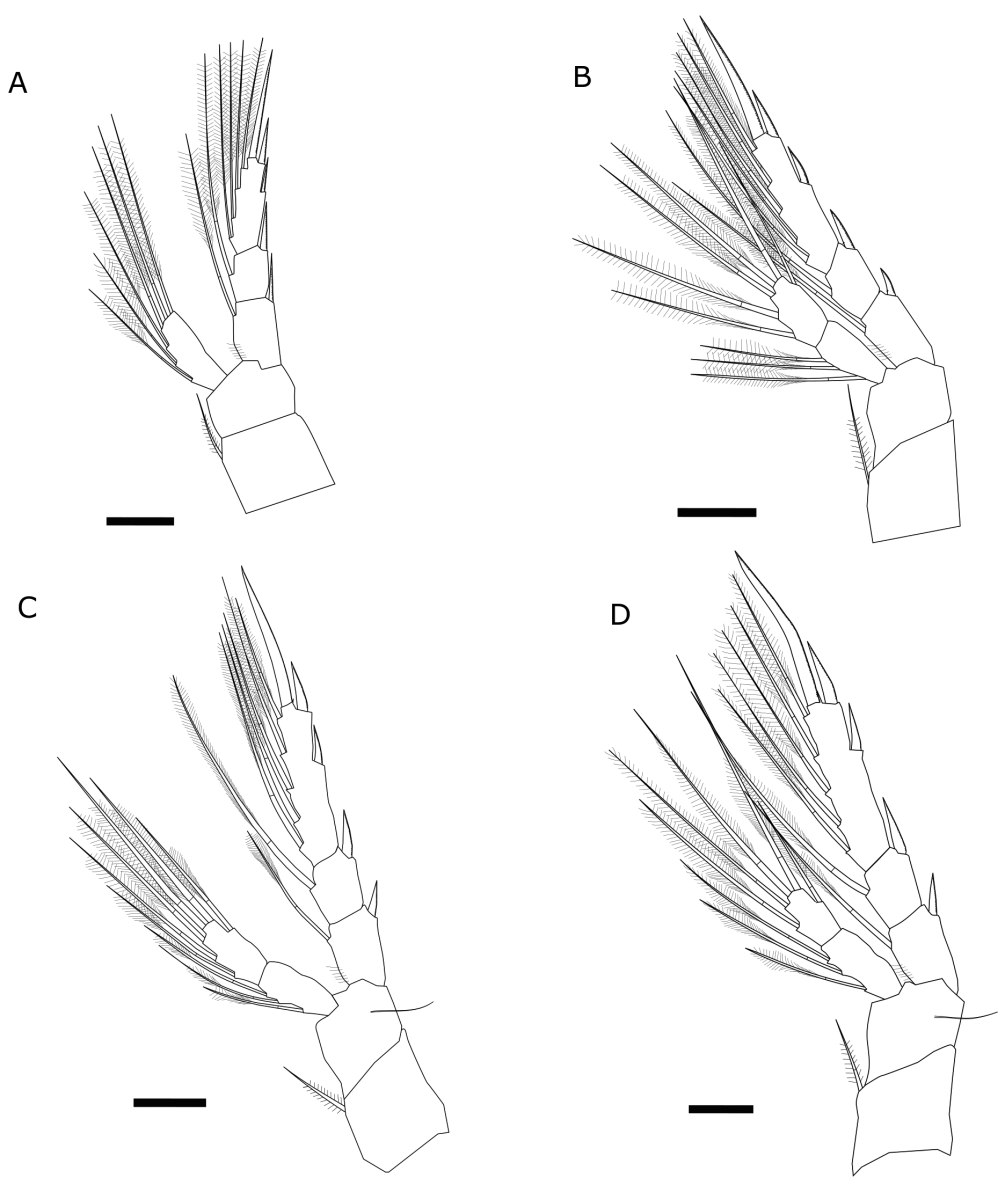
Legs of *Eurytemoralacustris***A** first pair **B** second pair **C** third pair **D** fourth pair. Scale bars: 20 µm.

**Figure 4.**
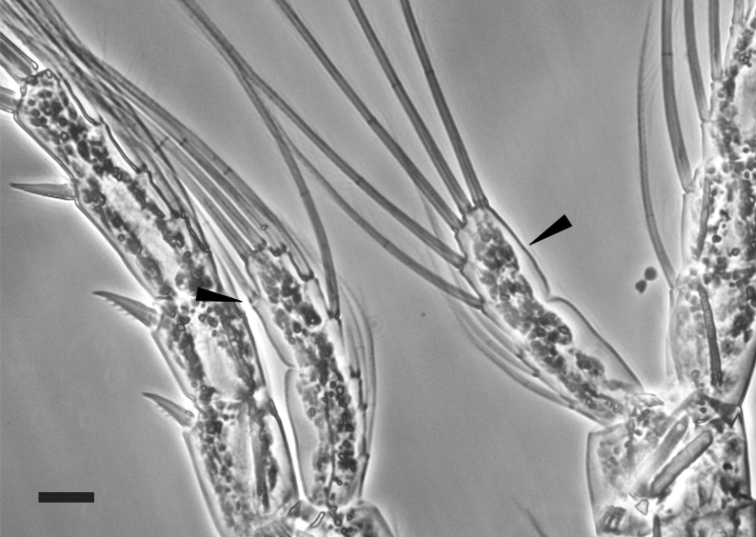
Variability of number of setae on second segment of endopod on second pair of male leg of *Eurytemoralacustris*. Scale bar: 20 µm.

**Figure 5.**
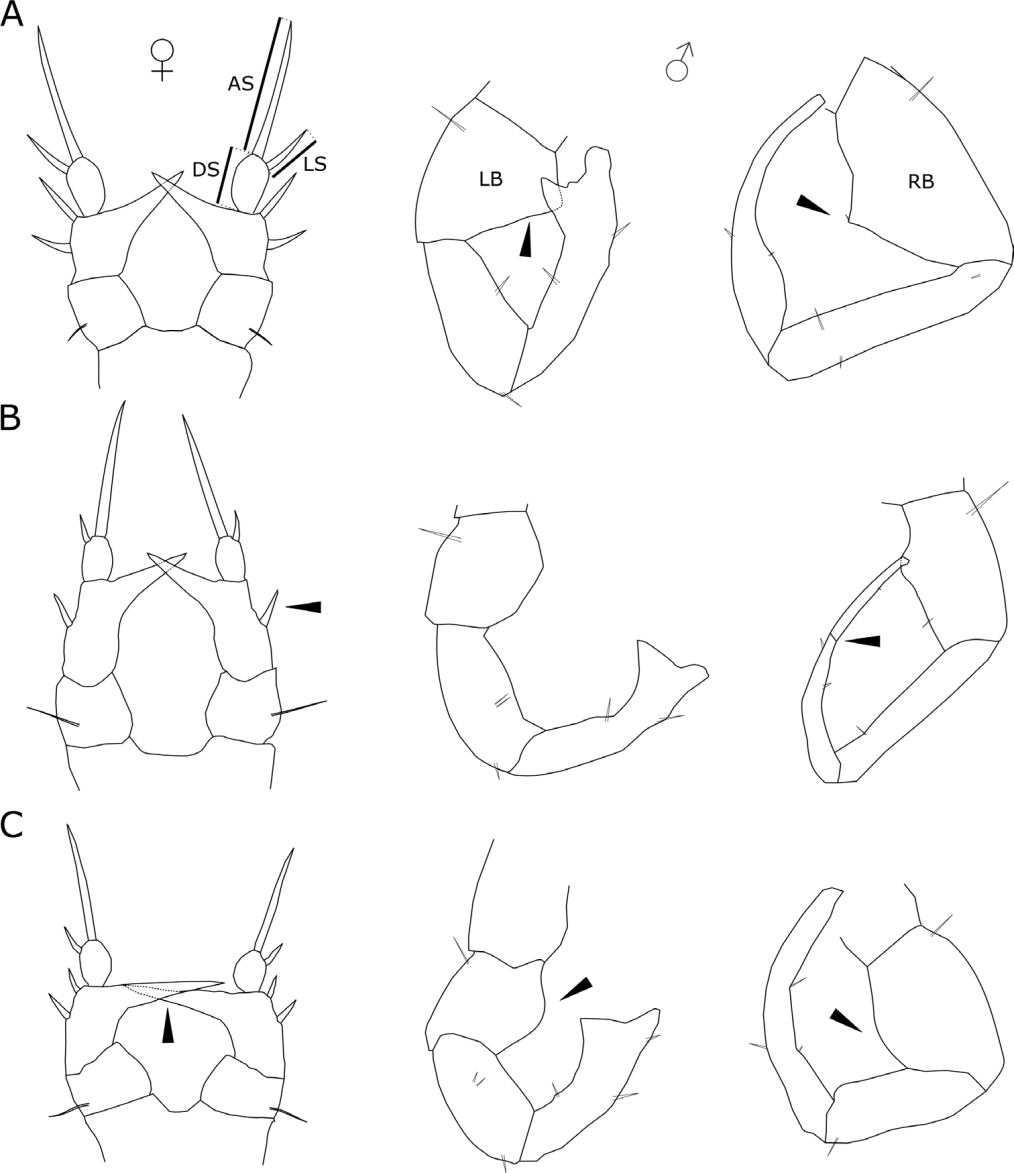
Fifth pair of legs of related *Eurytemora* species **A***E.affinis***B***E.velox***C***E.lacustris*. Abbreviations: AS – apical seta, DS – distal segment, LS – lateral spine, LB – left basipod, RB – right basipod. Arrows indicate important characteristics of fifth pair of legs.

First pair of legs (Fig. 3A) having coxa with medial seta. Exopod three-segmented with 2, 2, 7 setae. All segments with row of denticles toward distal edge. Endopod one-segmented with six setae. Second pair of legs (Fig. 3B) having coxa with medial seta. Exopod three-segmented, first and second segments each with one seta on the inner margin and one spine on the outer margin, third segment with five setae on inner margin, two spines on the outer margin and one terminal strong spine; first and third segments with row of denticles toward distal edge. Endopod two-segmented, first segment with three setae, second segment with six setae (adult male: endopod two- segmented; first segment with three setae, second segment with five or six setae Fig. 4). Third pair of legs (Fig. 3C) having coxa with medial seta. Exopod three-segmented; posterior face of the segments with denticles toward distal edge. Endopod two-segmented; posterior face of segments with denticles toward distal edge. Fourth pair of legs (Fig. 3D) having coxa with medial seta. Exopod three-segmented with 2, 2, 8 setae and spines; posterior face of segments with denticles toward distal edge. Endopod two-segmented with 3, 5 setae. Female fifth pair of legs three-segmented (Fig. 5C). Second segment with strong inner outgrowth and two spines, distal segment with long apical seta about four times longer than lateral spine. Distal segment about 1.5 length of lateral spine. Male fifth pair of legs three-segmented and asymmetric. Right leg basipodal segment with distally located smooth bulge on inner side about 1.2 times long as wide. Left leg basipod with smooth bulge on inner side as long as wide or slightly wider. The three most widely distributed species among the genus in Europe (*E.lacustris*, *E.affinis* and *E.velox*) were chosen for comparison of selected morphological characters. Females of *E.lacustris* and *E.affinis* are characterized by similar ratio of length to width of caudal rami (Tab. 1). *Eurytemoravelox* is characterized by a lower ratio of this parameter compared with females of *E.lacustris* and *E.affinis* (Tab. 1). In case of males, the differences of the discussed parameter were higher in each species considered; therefore, this parameter cannot be considered as indicative. Concerning the length to width ratio of the last endopod segment of the legs, *E.lacustris* shows the highest value among the three compared taxa (Tab. 1). In the case of the parameter referring to the ratio of the length of the last endopod segment to the length of the spine, there was greater variability.

**Table 1.** Selected characteristics of the three related *Eurytemora* species (*E.affinis*, *E.velox*, and *E.lacustris*). The value indicates the normalized ratio for size of the body parts.

	* E. affinis *			* E. velox *			* E. lacustris *			
	Sars 1895	Einsle 1993	Błędzki and Rybak 2016	Sars 1895	Einsle 1993	Błędzki and Rybak 2016	Sars 1895	Einsle 1993	Błędzki and Rybak 2016	present study
endopod 1 (length-width) ♀				100/36.1				100/33.7		100/31.9
endopod 1 (spine length-width) ♀				100/102				100/96.4		100/119
endopod 2 (length-width) ♀										100/24.2
endopod 2 (spine length-width) ♀										100/93.4
endopod 3 (length-width) ♀	100/24.6			100/39.8			100/21.8			100/21.6
endopod 3 (spine length-width) ♀	100/104			100/103.9			100/96.4			100/92.5
endopod 4 (length-width) ♀		100/31.9		100/35.3						100/24.9
endopod 4 (spine length-width) ♀		100/97.4		100/100						100/102
furca (length-width) ♂	100/8.1		100/11.4		100/16.3	100/12.3		100/14.6	100/10.6	100/14.2
furca (length-width) ♀	100/11.1	100/14	100/13.1	100/24.6	100/25.1	100/26.4	100/13.5	100/14.6	100/13.8	100/19
body length (µm)♂	1150	1200–1800	800–1900	2200	1300–2200	1100–2200	1300	1000–1500	1100–1500	1272–1456
body length(µm)♀		1100–1700	750–1650	1850	1000–1900	1500–1850		<1400	1000–1400	1274–1483

### Molecular analysis

**Figure 6.**
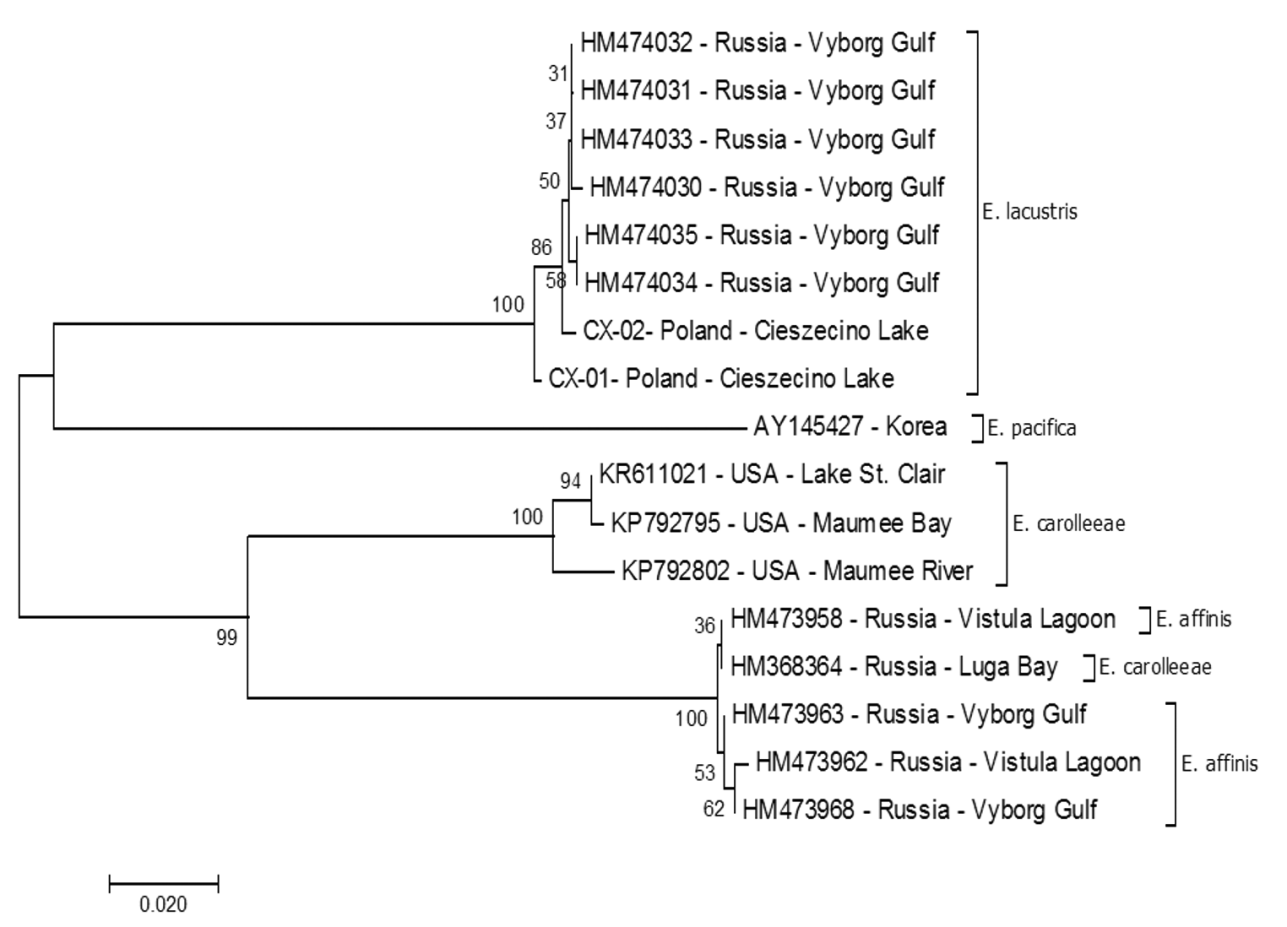
Evolutionary relationship of taxa based on *mCOI* sequences. The phylogenetic tree was inferred using the Minimum Evolution method (MEGA 10.0.5, Kumar et al. 2018). The evolutionary distances were computed using the Tamura 3-parameter method [3] and are in the units of the number of base substitutions per site. Sequences deposited at GenBank from Sukhikh et al. (2013, 2018), Vasquez et al. (2016), and Alekseev and Sukhikh (unpublished).

For each specimen of *E.lacustris*, amplicons of the expected length were obtained, of approximately 612 bp for the *COI* gene and 369 bp for the *cytb*. Subsequently, we performed nucleotide sequence analysis. *Cox1* gene analysis showed high degree of similarity in the nucleotide sequences for the population from Lake Cieszęcino (Poland). Two haplotypes were distinguished: one of them, described as CX-01 (MH316161) was represented only by 12.5% of the population (10 individuals). Sequencing of the *cox1* gene resulted in 612 bp, of which differences were related to the five nucleotide positions (Tab. 2). The four substitutions were transitions (3: A↔G and 1: T↔C), whereas one was transversion (A↔C). All substitutions were synonymous and they did not cause amino acid substitutions in the protein. The obtained sequences were compared to analogous sequences deposited in the GenBank, derived from *E.lacustris* from Russia (Table 3; Alekseev and Sukhikh, Russia, Baltic See, St Petersburg, Vyborg Gulf). In total, there were ten substitutions between the Polish and Russian sequences, which also had no consequences at the protein level. Calculations based on the Kimura-2-parameter model revealed distance ranging from 0.000-0.002 to 0.011 (CX-01 vs HM474030) and an overall distance of 0.005 (S.E. 0.001). An analysis of kinship (Fig. 6) was made using the nucleotide sequences of *E.lacustris* from Lake Cieszęcino and sequences of *E.lacustris* and related species (*E.affinis* and *E.carolleeae*) caught in Russia (GenBank). All nucleotide sequences of the *COI* gene form a separate clad, with the sequence from Poland designated as CX-01 (MH316160) by us as the initial for them. Molecular analysis based on *COI* showed that our collection belongs to the same species of *E.lacustris* inhabiting the waters of the Baltic Sea.

**Table 2.** Variability in the nucleotide sequences of the gene *cox1* for *Eurytemoralacustris* from Cieszęcino Lake (NW Poland) and Baltic Sea, Vyborg Gulf (Alekseev and Sukhikh, unpublished).

**Position in sequences/ Accession number**	**58**	**69**	**210**	**303**	**309**	**399**	**405**	**759**	**582**	**612**
	T	T	G	A	C	A	A	T	T	C
CX-02*	·	·	·	·	·	·	·	·	·	·
CX-01**	·	·	A	G	A	G	·	C	·	-
HM474035	C	·	A	·	·	·	G	·	·	T
HM474034	C	·	A	·	·	·	·	·	·	T
HM474033	·	·	A	·	·	·	·	·	·	·
HM474032	·	·	A	·	·	·	·	·	·	T
HM474031	·	·	A	·	·	·	·	C	·	T
HM474030	·	C	A	·	·	·	·	C	C	T
SUBSTITUTION	TZ	TZ	TZ	TZ	TW	TZ	TZ	TZ	TZ	TZ

CX-02*(GenBank accession number: MH316160), CX-01** (MH316161); TZ-transition, TW-transversion;

**Table 3.** The most commonly used markers in the determination of selected Copepoda species.

**Species**	***COI***	***cytb***	***rRNA* genes**	**TGDNA**	**ISSR-PCR**	**Authors**
*Calanusfinmarchicus*, *C.glacialis*, *C.helgolandicus*, *Neocalanuscristatus*, *N.flemingeri*, *N.plumchrus*, *Pseudocalanusmoultoni*, *P.newmani*	×					Bucklin et al. 1999
* Leptocaris canariensis *	×			×		Fazhan et al. 2016; Waiho et al. 2013
* Leptodiaptomus garciai *	×					Montiel-Martínez et al. 2008; Ortega-Mayagoitia et al. 2018
*L.minutus*, *Onychodiaptomussanguineus*	×					Thum and Derry 2008
* Pseudocalanus moultoni *	×					Aarbakke et al. 2011
* Skistodiaptomus pallidus *	×	×				Thum and Derry 2008
* S. reighardi *	×					Thum and Derry 2008
*S.oregonensis*, *S.pygmaeus*	×	×	×			Thum and Harrison 2009
* Tigriopus japonicus *			×			Ki et al. 2009
Species of family Temoridae incl. *T.discaudata*, *T.longicornis*	×	×	×	×		Blanco-Bercial et al. 2011, 2014; Khodami et al. 2017
* Eurytemora affinis *					×	Gasmi et al. 2014
* Eurytemora affinis *	×					Gorokhova et al. 2013; Sukhikh et al. 2013; Vasquez et al. 2016
* Eurytemora carolleeae *	×					Sukhikh et al. 2013; Vasquez et al. 2016
* Eurytemora lacustris *	×					GenBank Alekseev and Sukhikh, unpublished
* Eurytemora lacustris *	×	×				Present study

Analysis of the *cytb* gene sequence from the *E.lacustris* population from Lake Cieszęcino revealed three haplotypes: designated as CY-01*** (MH316162); CY-02** (MH316163), and CY-03* (MH316164). These haplotypes were determined based on the analysis of substitution in the nucleotide sequence, in which three substitutions (transitions) were observed on the 369 bp section, at positions 205, 211, and 316 (Tab. 4). Overall mean distance was 0.005. None of the changes caused substitution of amino acids in the protein. Due to the lack of analogous sequences in GenBank of the *cytb* gene (the reported sequences are the first for the genus *Eurytemora*), it was not possible to perform similarity analysis within the species using that gene, as well as phylogenetic analysis between closely related species.

**Table 4.** Variability in the nucleotide sequences of the gene *cytb* for *Eurytemoralacustris* from Lake Cieszęcino, NW Poland.

**Position in sequences/ Accession Number**	**205**	**211**	**316**
	**T**	**G**	**C**
CY-03*	·	·	T
CY-02**	·	·	·
CY-01***	C	A	·

CY-01*** (GenBank accession number: MH316162); CY-02** (MH316163); CY-03*(MH316164).

## Discussion

The occurrence of *E.lacustris* in the Lake Cieszęcino has not been recorded so far (NW Poland). The lake in which the new record of the species was obtained is one of the smallest in terms of surface area (Sars 1895; Czeczuga 1960; Kasprzak et al. 2005; Maier et al. 2011; Karpowicz and Kalinowska 2018). *Eurytemoralacustris* does not have the ability to produce resting eggs and was not observed in the river network upstream and downstream. Therefore, we assume that these are stable populations in this lake. Another location of occurrence of this species indicates that it is more widespread species in the lakes of Western Pomerania (Poland) than previously assumed. Presently, *E.lacustris* is recorded in at least six West Pomeranian lakes (Lakes Cieszęcino, Drawsko, Ińsko, Lubie, Siecino, and Żerdno).

### Morphology

*Eurytemoralacustris* and *E.affinis* are similar in morphological terms. Sars (1895) described the genus *Eurytemora* and indicated that except to the construction of the fifth pair of legs on both species and wings on the fifth thoracic segment on *E.affinis* on females, both taxa are very similar. Therefore, particularly for younger specimens with fifth pair of not fully developed legs, these species can be misidentified. Hence *E.lacustris* has been sometimes reported in the typical habitats of *E.affinis*, i.e., in the estuaries of the southern Baltic.

Swimming legs may not differ on the number of setae on female and male individuals (except P2 endopod 2). It is not yet determined whether this phenomenon is specific for the population from Cieszęcino Lake, or whether it is common for that species. Gaviria and Forró (2000) studying the populations of *E.velox* (Lilljeborg, 1853) of Europe showed changes in the leg spinulation pattern of some individuals of the Austrian and Hungarian populations. They stated that a possible explanation could include effects of pollution (Gaviria and Forró 2000). The catchment of Cieszęcino Lake is anthropogenically less transformed, which allows us to suppose that there is no excessive pollution of the lake’s waters; hence, the polymorphism observed within this species is probably not the result of anthropogenic changes.

The parameters of some appendages differ between related species (*E.lacustris*, *E.affinis* and *E.velox*). At the same time, differences are also observed between different authors. However, the parameter concerning the width-to-length ratio of the last endopod segment in females indicates variation between the indicated species (*E.lacustris*, *E.velox*, and *E.affinis*). Therefore, this parameter seems to be able to serve as an auxiliary parameter in morphological identification.

The most indicative features among *Eurytemora* species are related to fifth pairs of legs (Alekseev and Souissi 2011). Female of *E.lacustris* has strong inner outgrowth almost at right angle in relation to the basipodite. Two spines on second segment distinguish *E.lacustris* from *E.velox*, that has one spine (Fig. 5A–C). However, Gaviria and Forró (2000) found variability in the number of spines on female first exopod segments. Female (*E.lacustris*) distal segment with long apical seta about four times longer than lateral spine, while at *E.affinis* lateral spine is less than three times long as apical seta. Male *E.lacustris* fifth pair of legs three-segmented what distinguish that species from *E.velox* that has four segments at right leg. Legs basipod segments with smooth bulges on inner side distinguish *E.lacustris* from *E.velox* and *E.affinis* that have sharp shape basipodal inner side.

### Molecular analysis

Morphology is the primary criterion in the determination of species affiliation. However, the small size of copepods could be an obstacle in terms of morphological determination. Genetic analysis among such species allowed to verify the species within morphologically similar specimens and hence give indication of cryptic species (Lee 2000; Lajus et al. 2015). Sometimes morphological differences are imperceptible and are not indicated on taxonomic keys. Therefore, collection of molecular data allows the identification of species (Thum 2006). The invasion of the American species *E.carolleeae* in the Gulf of Finland was found through morphological studies, as well as through molecular genetic tools (Sukhikh et al. 2013). In addition, the analysis of morphological and molecular features allows to determine whether the methods of morphological identification include those features that are important to identify species (Thum and Harrison 2008).

*COI* is a unique diagnostic tool for identifying copepods at the species level with barcoding (Bucklin et al. 2003; Costa et al. 2007; Sukhikh et al. 2013; Blanco-Bercial et al. 2014; Sukhikh et al. 2018). Costa et al. (2007) noted that the level of genetic variation between congeneric species on crustaceans is 17.16% on average, which is extremely high compared to variation in other groups of animals. In addition, the level of intraspecific variability in crustaceans (average 0.46%) is only slightly higher than in other groups of animals. According to our results, the average variation between Polish and Russian populations of *E.lacustris* is 0.5%, which would confirm the hypothesis of Costa et al. (2007) about intraspecific variability on crustaceans. At the same time, between the compared species of *Eurytemora*, the genetic distance is high with ranges from 23.1% between *E.affinis* and *E.lacustris*, to 21.3% between *E.carolleeae* and *E.lacustris* and to 13.6% between *E.carolleeae* and *E.affinis*.

Sars (1895) stated that *E.lacustris* is “a perfectly freshwater” species. Nevertheless, there are cases where *E.lacustris* is recorded in brackish waters (Vekhov 2001; Sukhikh et al. 2019). This is most likely a result of drifting of individuals from lakes that are relatively close to estuarine waters. The drift can be particularly effective during the night when low intensity of light limits the pressure of the planktivorous fish on drifting plankton (Czerniawski et al. 2016). Probably in this way, individuals of *E.lacustris* drifting from Lake Ladoga arrived at Vyborg Gulf. Hence, Sukhikh et al. (2019) indicated Vyborg Gulf as the location for obtaining individuals of *E.lacustris*. Our research confirms that this is the same species. However, the question is whether populations that reach the bay acquire the ability to survive in low-salinity waters, which would be the beginning of speciation of population entering the waters with low salinity, such as the Gulf of Finland. We can state that *E.lacustris* is not a purely freshwater species as Sars (1895) and others believed. Further research should be conducted to explain this phenomenon.

*Cytb* also belongs to conservative mitochondrial genes that are successfully used to identify species of vertebrates (Lopez-Oceja et al. 2016) and invertebrates (Merritt et al. 1998; Thum and Derry 2008; Thum and Harrison 2008; Blanco-Bercial et al. 2014). In previous studies on *Eurytemora*, no data was found on *cytb* analysis. The *cytb* sequences reported by us are the first for this genus; hence, the comparison to the closest related species was not possible.

The use of molecular and morphological analyses allows for a reliable determination of species affiliation and further research. The identification of juvenile stages is possible primarily with molecular analysis. Bucklin et al. (2003) also suggested the use of molecular methods to analyse unsorted zooplankton to determine species affiliation. When analysing the genetic structure and phenotypic traits of *E.lacustris*, we obtained data that may be useful for further monitoring of this species and for research concerning the origin of individual populations. Accurate taxonomic identification of species at all stages of life is crucial for understanding and predicting processes that determine the dynamics of planktonic communities.
